# Why organisational diversity matters in a circular economy and society

**DOI:** 10.1016/j.resconrec.2026.108833

**Published:** 2026-04-01

**Authors:** Wim Van Opstal, Nancy Bocken, Jan Brusselaers

**Affiliations:** a Unit of Sustainable Materials and Chemistry, VITO, Boeretang 200, 2400 Mol, Belgium; b Maastricht Sustainability Institute, School of Business and Economics, Maastricht University, Tapijn 11 Building D, P.O. Box 616, 6200 MD Maastricht, the Netherlands; c Institute for Environmental Studies, VU Amsterdam, De Boelelaan 1111, 1091 HV Amsterdam, the Netherlands; d Engineering Management, Faculty of Business and Economics, University of Antwerp, Prinsstraat 13, 2000 Antwerpen, Belgium

A successful transition to a circular economy (CE) relies on the interplay of diverse organisational forms – such as firms, cooperatives, non-profit associations, and public agencies – each offering distinct capabilities to address its challenges. Academic and policy debates often reduce CE governance to a binary lens that positions firms as innovators and governments as regulators, narrowing the analytical focus to market incentives and regulation.

The CE is commonly defined as an economic system that minimises waste and resource use by reducing, reusing, recycling, and recovering materials across production, distribution, and consumption (Kirchherr et al., 2017). It seeks to decouple economic activity from the consumption of finite resources and to design systems that maintain material value for as long as possible (Geissdoerfer et al., 2017). A CE entails the deliberate design of industrial and societal systems that narrow, slow, close, and regenerate material and energy flows (Konietzko et al., 2020). The complementary notion of a *circular society* extends the focus beyond resource efficiency to include social relations, governance processes, and justice dimensions. It emphasises that circular transitions must ensure equity, participation, and recognition in how resources and responsibilities are organised (Calisto Friant et al., 2023; Jaeger-Erben et al., 2021). A circular society thus embeds the circular economy within a wider social and institutional context, acknowledging that social innovation, citizen engagement, and shared value creation are essential conditions for systemic change.

While regulative, normative, and cultural-cognitive pillars jointly define the institutional environment for a circular economy and society, much of the academic and policy discourse remains focused on governments and firms. This institutional bias overlooks the broader coalition of actors required to sustain systemic change. A genuinely circular society depends on the interplay between multiple institutional logics and organisational forms that operate beyond a binary firm-state lens. This calls for a closer examination of the *third sector* – civil society organisations situated between the market and the state, encompassing activities undertaken by non-governmental and voluntary organisations (Evers and Laville, 2004). These organisational forms involve cooperatives, non-profit associations, and social enterprises, which bring distinct capabilities, incentives, and forms of participation that can complement regulatory authority and market-based innovation.^1^

Three interrelated challenges clarify why organisational diversity matters in circular transitions. *First*, the transition faces market failures that firms and states cannot always address efficiently. Externalities arise when markets fail to price environmental and social costs and benefits. Information asymmetries limit reliable knowledge about product quality, environmental performance, and long-term value. Split incentives arise when costs and benefits accrue to different actors, such as investors and users. Within organisations, split incentives create agency problems, where delegated decision-makers do not fully internalise organisational objectives. Transaction costs relate to time, effort, and resources required to search for partners, negotiate contracts, coordinate activities, and monitor compliance, which can discourage the implementation of circular strategies. Trust matters across these failures because it reduces uncertainty and coordination costs, while a lack of trust amplifies barriers and slows the adoption of circular strategies. *Second*, calls for a “circular society” (Jaeger-Erben et al., 2021) and a “just circular economy” (Kirchherr, 2021) emphasise the need to integrate equity, participation, and recognition, which often fall outside the incentive structures and decision logics of both firms and state bureaucracies. *Third*, circularity does not merely alter production processes and value chains; it reconfigures social institutions and governance mechanisms that structure economic interaction. It relies on novel but long-term relationships: between producers and users, across generations, and between formal and informal sectors. These require capacities to relate, resonate, and responsibilise – referring to the ability to build and sustain relationships, align values and meanings, and support shared responsibility over time – which are unevenly distributed across organisational forms and governance arrangements (Jaeger-Erben et al., 2025).

Organisational forms are institutional arrangements through which economic activities are structured, governed, and legitimated (Ben-Ner, 2018). They comprise rules and norms governing ownership, accountability, control rights, incentive structures, and decision-making. Organisational forms describe governance and ownership structures rather than e.g., environmental orientations, which can vary widely within each category, including among start-ups and incumbent firms. This distinction matters, as literature on hybrid organisations has mainly focused on purpose hybridity, examining how organisations combine social and commercial missions (Alter, 2007). These purpose hybridity frameworks describe organisational forms along a spectrum between social value creation and financial return. However, no organisational form inherently pursues circular strategies, and engagement with circularity depends on internal incentives and priorities, sectoral conditions, and regulatory and market signals. To understand boundary conditions for a circular transition, the question is not only *why* organisations act, but also *how* they are structured to pursue circularity effectively while addressing market failures, relational complexity, and normative goals.

We propose a complementary perspective that treats organisational forms as institutional instruments with distinct comparative advantages in enabling and embedding CE transitions, as summarised in Fig. 1.

**Fig. 1:**
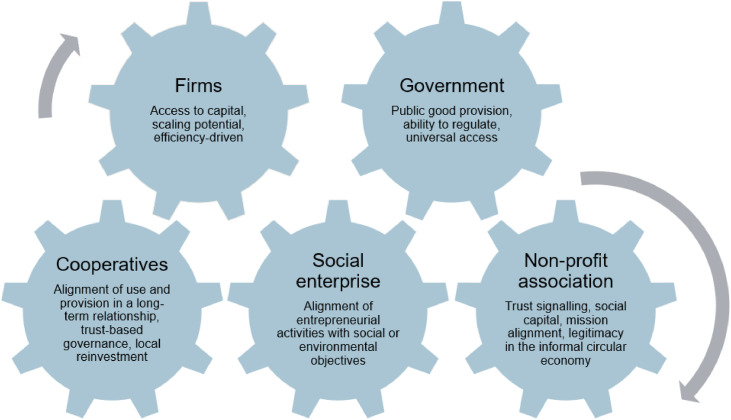
Comparative organisational advantages in addressing circular economy challenges.

*Investor-owned firms* benefit from clearly defined property rights, access to capital markets, and incentives aligned with profit and efficiency. These features support innovation and scaling and can drive CE adoption when aligned with regulation and consumer demand, for example through remanufacturing, product-as-a-service models, or take-back schemes in manufacturing and retail sectors. However, these same features often discourage investment in durability, repairability, or reuse – especially when returns accrue to others. Profit imperatives can lead to cost externalisation, and firms may struggle with trust-building, community alignment, or collective action when returns are diffuse or uncertain.

*Governments* possess the authority and resources to enforce regulation, redistribute benefits, and provide public goods. They can correct market failures through standards, coordination, and subsidies, and enable innovation by temporally sheltering promising circular initiatives from market pressures or investing in high-risk, high-potential solutions. Examples include regulatory frameworks like the EU Circular Economy Action Plan, municipal support for repair cafés, and the provision of semi-public goods such as waste management. However, governments often suffer from bureaucratic rigidity and limited responsiveness to decentralised CE initiatives. State-led interventions may also lack participatory mechanisms to build trust and engage communities.

*Cooperatives* provide a first illustrative alternative for the firm-state binary. Cooperatives emerged to address common-pool resource problems and foster community resilience. They enable value to be shared among members and reinvested locally, rather than extracted by shareholders. By aligning users and producers in long-term relationships, cooperatives help to internalise trust, reduce transaction costs, and enable self-enforced agreements through participatory governance. Shared ownership and voluntary engagement enhance social acceptance, reduce asymmetric information, and embed circularity in locally rooted infrastructures – as seen in many renewable energy source cooperatives. However, democratic governance increases decision-making costs and may lead to free-riding or demutualisation, especially with growing or heterogeneous membership. Access to capital is also constrained by limits on profit distribution, and cooperatives often lack cognitive and normative legitimacy in regulatory and financial systems.

*Non-profit associations* are typically defined as organised, private, self-governing and non-profit-distributing entities with a meaningful degree of voluntary participation. Across the world, they are mostly recognised as voluntary associations formed to pursue public-benefit or mutual-benefit aims under the freedom of association, with a legal non-distribution constraint that prohibits the allocation of surplus to founders, members or directors. This reduces profit-maximisation incentives, mitigates agency problems, and signals trustworthiness – attracting mission-oriented agents and enabling long-term engagement. These traits enable them to address externalities, engage volunteers, and support collective action. Beyond filling institutional gaps, non-profits reconfigure governance by mobilising trust, reciprocity, and identity, especially when market signals fail to reflect social value. In informal repair and reuse economies, non-profit associations can legitimise marginalised actors, diffuse learning, and amplify political voice through translocal networks. However, they face constraints. They often rely on volunteer labour, project-based funding, and mission legitimacy, which can limit scalability, continuity, and responsiveness to changing societal demands. Internal governance structures may also reproduce exclusionary norms or paternalistic dynamics, undermining their inclusive potential.

*Social enterprises* can be defined as organisations that pursue a social mission through entrepreneurial means, combining market-oriented activities with explicit social or environmental goals (European Commission, 2020). *Social enterprises* illustrate hybrid organisations combining entrepreneurial activity with social objectives. Positioned between markets and public support, they seek to generate positive externalities, such as employment for people distant from the labour market – as in Work Integration Social Enterprises (WISEs) – or the provision of affordable repair and reuse services. WISEs frequently engage in repair, reuse, and recycling, combining ecological and social goals. Yet social enterprises also face pressure from market-based funding and performance metrics that neglect social outcomes or long-term value creation.

Each organisational form offers comparative advantages for addressing specific CE challenges. Externalities, transaction costs, and public goods dilemmas call for institutions that align incentives, build trust, and enable long-term cooperation. Property rights, governance mechanisms, and contracts influence organisational behaviour. Relational contracts – informal, self-enforcing agreements sustained through repeated interaction and shared expectations rather than detailed external enforcement – thrive in cooperative and associative settings where repeated interaction, shared norms, and mission alignment reduce enforcement costs. Game-theoretic insights show that collective action problems can be mitigated through communication, identity, and reputation – conditions more common in non-profit associations and cooperatives than in anonymous market transactions.

These same organisational dynamics also have implications for a just circular transition (Kirchherr, 2021). They shape who participates in circular activities, who gains or loses from circular value creation, and whose voices are recognised in governance processes. Non-profit associations make visible the knowledge and contributions of informal workers often overlooked in mainstream CE strategies. Cooperatives empower communities to retain agency over resources and decisions. Social enterprises offer pathways for labour market inclusion alongside CE goals. Together, these organisations not only support circular material flows, but also reconfigure power relations, institutions, and values.

A successful circular transition relies not on any single organisational form, but on their *interplay*. Each brings distinct capabilities, yet it is their combination that enables systemic change. Governments create enabling conditions for a circular economy and society through regulation, investment, and coordination, but their impact deepens when partnering with trusted intermediaries such as non-profit associations that mobilise citizens. Investor-owned firms are essential for innovation and scaling, yet their efforts can be grounded and legitimised through collaboration with cooperatives that embed circularity locally. Social enterprises often operate at the interface of market and mission, linking vulnerable groups to circular activities like repair or reuse, but their effectiveness improves when supported by public procurement or by networks facilitated by non-profits. This interplay enables diverse organisational logics to co-evolve: state agencies provide stability, firms offer dynamism, cooperatives foster trust and shared ownership, and associations and social enterprises build inclusion and resilience. Therefore, we invite policymakers, scholars, and practitioners to consider how organisational diversity, their comparative advantages, and their interplay can broaden CE strategies and policies, making them not only more effective, but also more equitable and inclusive. This perspective can also enable active support for higher-order circular strategies that deliver substantial systemic benefits but remain under-implemented due to market barriers.

## CRediT authorship contribution statement

**Wim Van Opstal:** Writing – review & editing, Writing – original draft, Visualization, Validation, Methodology, Investigation, Formal analysis, Conceptualization. **Nancy Bocken:** Writing – review & editing, Validation. **Jan Brusselaers:** Writing – review & editing, Validation.

## Declaration of competing interest

The authors declare that they have no known competing financial interests or personal relationships that could have appeared to influence the work reported in this paper.

## Data Availability

No data was used for the research described in the article.

## Footnotes

1 Other third-sector forms include foundations and mutualities. In the context of a CE they appear mainly as indirect enablers rather than operational actors. Foundations tend to fund, convene and diffuse knowledge (e.g., the Ellen MacArthur Foundation) – rather than deliver circular services themselves. Mutualities are health-insurance bodies whose statutory mandate lies outside CE activities.
